# A Perspective on How Fibrinaloid Microclots and Platelet Pathology May be Applied in Clinical Investigations

**DOI:** 10.1055/s-0043-1774796

**Published:** 2023-09-25

**Authors:** Etheresia Pretorius, Douglas B. Kell

**Affiliations:** 1 Department of Physiological Sciences, Faculty of Science, Stellenbosch University, Stellenbosch, Matieland, South Africa; 2 Department of Biochemistry and Systems Biology, Institute of Systems, Molecular and Integrative Biology, Faculty of Health and Life Sciences, University of Liverpool, Liverpool, United Kingdom; 3 The Novo Nordisk Foundation Centre for Biosustainability, Technical University of Denmark, Lyngby, Denmark

**Keywords:** microclots, platelet hyperactivation, inflammatory molecules, vascular complications

## Abstract

Microscopy imaging has enabled us to establish the presence of fibrin(ogen) amyloid (fibrinaloid) microclots in a range of chronic, inflammatory diseases. Microclots may also be induced by a variety of purified substances, often at very low concentrations. These molecules include bacterial inflammagens, serum amyloid A, and the S1 spike protein of severe acute respiratory syndrome coronavirus 2. Here, we explore which of the properties of these microclots might be used to contribute to differential clinical diagnoses and prognoses of the various diseases with which they may be associated. Such properties include distributions in their size and number before and after the addition of exogenous thrombin, their spectral properties, the diameter of the fibers of which they are made, their resistance to proteolysis by various proteases, their cross-seeding ability, and the concentration dependence of their ability to bind small molecules including fluorogenic amyloid stains. Measuring these microclot parameters, together with microscopy imaging itself, along with methodologies like proteomics and imaging flow cytometry, as well as more conventional assays such as those for cytokines, might open up the possibility of a much finer use of these microclot properties in generative methods for a future where personalized medicine will be standard procedures in all clotting pathology disease diagnoses.

The processes of normal blood clotting are well established (for example 1 2 3 4 5 ). The terminal steps involve the self-assembly of fibrinogen molecules that have been cleaved by thrombin (which removes two “fibrinopeptides” 6 7 8 ) into long fibers with a diameter typically in the range of 50 to 100 nm. Some time ago, we established that blood frequently clotted into anomalous forms, referred to in earlier papers as “dense matted deposits” 9 10 11 12 , that were also rather resistant to fibrinolysis (proteolysis). 13 It was subsequently recognized 14 that this anomalous form was in fact amyloid in character 15 and could be stained with well-established, fluorogenic amyloid stains such as thioflavin T 15 16 17 18 19 20 21 22 or the oligothiophenes marketed as “Amytracker” dyes. 16 19 21 We used fluorescence microscopy imaging as our method of choice. To distinguish them from the fibrils in established amyloidosis 23 (commonly with diameters less than 15 nm 24 25 ) and to recognize that they consist mainly of fibrin aggregates (plus other trapped molecules) in the range of 2 to 200 μm and with individual fibers with diameters commonly in the range of 50 to 100 nm or more, we refer to them as fibrinaloid microclots. 17 Such fibrinaloid microclots or fibrin(ogen) aggregates, which “spontaneously” form in the circulation (presumably under the action of thrombin'), have recently been reported in the plasma of patients with type 2 diabetes mellitus (T2DM), 19 26 in those with acute coronavirus disease 2019 (COVID-19) 26 27 28 29 30 and in particular in those with persistent symptoms related to postacute sequelae of COVID (PASC), more commonly known 31 32 33 as long COVID. 17 34 35

In addition, platelets in the hematocrit and whole blood of participants with various inflammatory conditions are well known to be hyperactivated. 27 36 37 38 39 40 41 42 For these studies, we also used fluorescence microscopy imaging as our method of choice for platelet imaging. (After centrifuging whole blood, there are still platelets present in the hematocrit). These platelets might in fact be considered as those that were most fragile in vivo. These microclots and hyperactivated platelets have been implicated in the thrombotic and systemic inflammatory complications of various diseases. In addition, numerous well-known inflammatory molecules have been found trapped inside these insoluble microclots present in acute COVID-19 and long COVID. 17 35 These particular microclots (whose formation can be catalyzed by the severe acute respiratory syndrome coronavirus 2 [SARS-CoV-2] S1 spike protein) 34 are very resistant to digestion protocols. 13 15 35 However, microclots from T2DM (and the few that are always present in plasma samples of healthy participants) digest easily with standard protein digestion protocols. Important questions from these differential results are raised regarding the pathophysiological relevance of microclots and platelet hyperactivation. These findings warrant a discussion to determine if these pathologies are indeed a cause or a consequence of disease and if they are indeed a predictor of clinical thrombosis or contributors to disease pathologies noted in the patient. Further, if these microclots and activated platelets are indeed a predictor of clinical symptoms, including patient disease progression and, in particular, thrombotic endothelialitis, could they ultimately then also become a microscopy imaging monitoring tool to be used to assess the effectiveness and progress of treatment regimens? Finally, an arising question is whether these variables are just innocent bystanders, albeit present in all inflammatory conditions, or a significant element of the etiology of these diseases. In the latter case, one may anticipate significant relationships between the nature and extent of fibrinaloid microclot formation and the severity of the disease. In addition, variants such as omicron that are far less virulent (albeit more transmissible) are—as we have recently shown 43 —far less able to cause microclots. This implies strongly that the microclots are part of the disease pathway. If the latter is in fact occurring, then a naive view might be that all microclots are “the same” and that platelet hyperactivation is universally similar in all conditions. As with any quantitative determination (like microscopy imaging), this is almost certainly not true, and the purpose of the present commentary is to rehearse the evidence for the kinds of differences that are either known or may be anticipated. We also point out the usefulness of microscopy imaging, as such a method allows for a fast and reliable snapshot of clotting pathology, to study both microclot formation and platelet hyperactivation.

## The General Pathway of Inflammation, Platelet Hyperactivation, and Microclot Formation in Chronic Disease

**Fig. 1:**
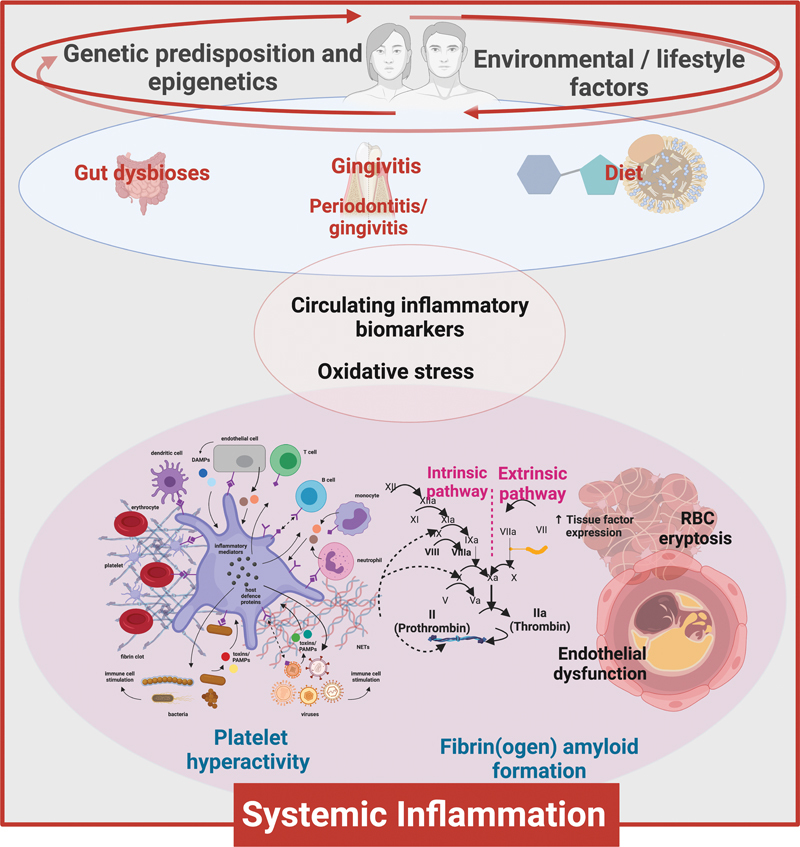
Visualizing various factors that influence disease to the understand the phenomena of the disease due to the response to an external stimulus 44 such as an infection. Figure created by authors using Biorender.com.

Fig. 1 shows the broad swathe of phenomena on which we focus, as they occur in response to an external stimulus 44 such as an infection. It recognizes that a chief set of pathways necessarily involves an external event—commonly an infection—that leads to inflammation (assessed via increases in the levels of inflammatory cytokines) and directly or indirectly to the formation of microclots and the hyperactivation of platelets. These phenomena can then feedback in a kind of autocatalytic cycle (in very unfavorable cases leading to a “cytokine storm” 45 46 ).

## Variations in Patterns of Cytokines

The presence and concentration of molecules, 47 including pro- (and anti-) inflammatory molecules vary considerably between individuals, even in “health” 48 49 50 51 , not least because of variations in age 52 and in the gut microbiome. 53 Considerable variation similarly exists in those with the “same” disease, 54 such as an infection 55 (including by SARS-CoV-2 56 57 58 59 60 ), the patterns often correlating with severity and/or outcome. The same is true in myalgic encephalitis/chronic fatigue syndrome 61 and long COVID. 62 Consequently, the presence of a soup of inflammatory molecules in circulation that individually might bind to the numerous receptors on platelets, and have direct protein–protein interactions, may therefore be a crucial predictor of the detailed effects of any systemic inflammation. These interactions should be seen as central to the cause and effect of disease development and presentation of both diverse and overlapping symptoms. Clotting and platelet pathologies should therefore not simply be seen as a predictor of clinical thrombosis. Such assumption would be oversimplifying the pathophysiological value of both identifying and studying the underlying causes of the presence of the inflammatory molecules in circulation.

## Differences in the Extent of Platelet Activation

**Fig. 2:**
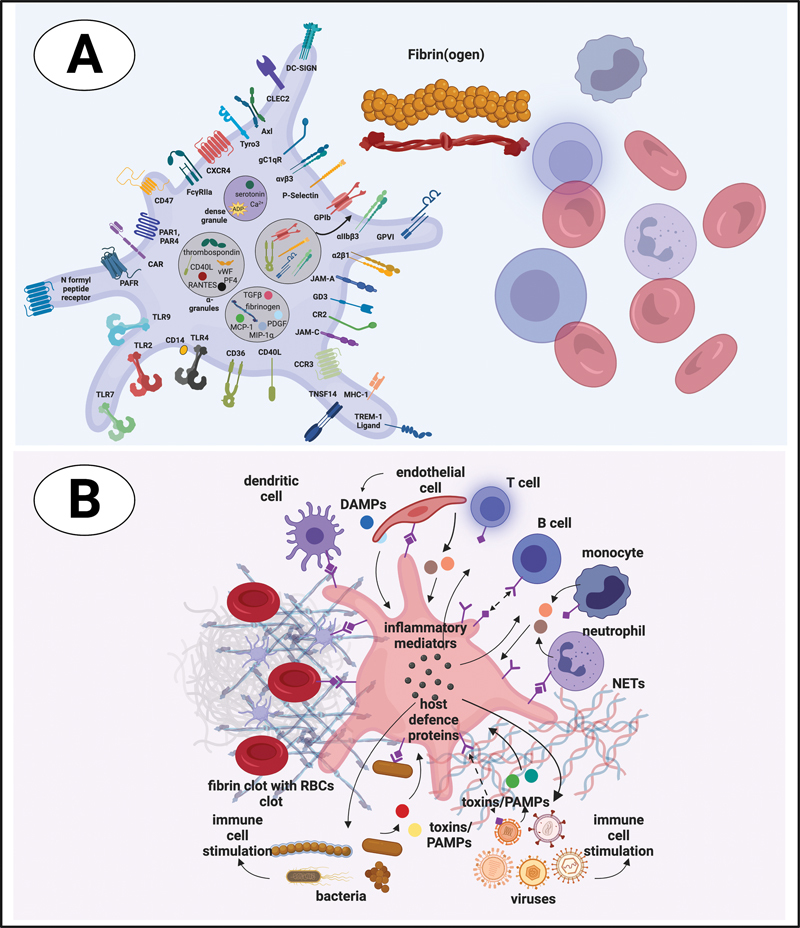
Platelet receptors and interactions with cells and proteins in circulation. Figure created by authors using Biorender.com.

**Fig. 3:**
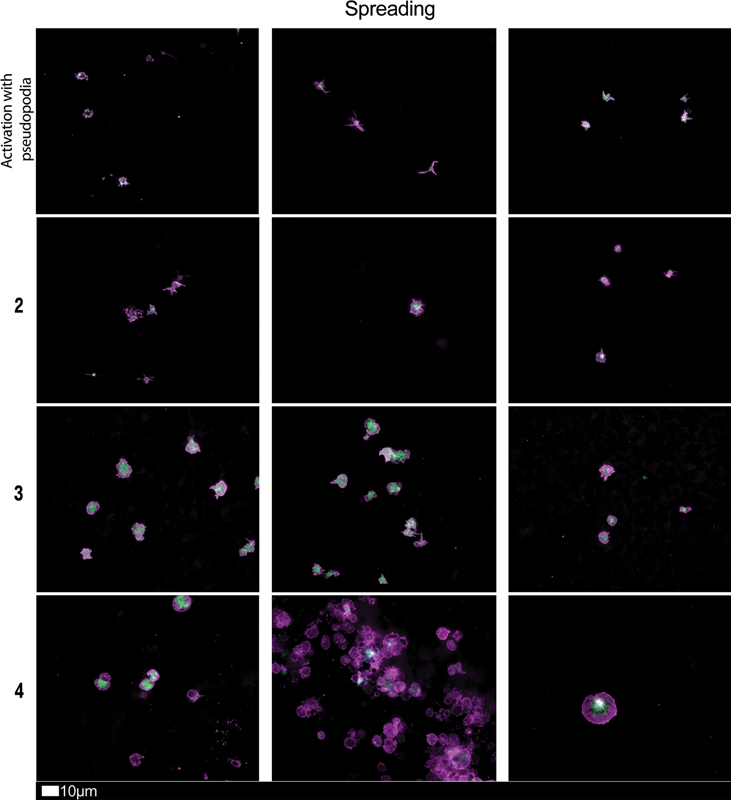
Fluorescence microscopy imaging examples of the different stages of platelet activation and spreading that was used to score platelet activation in long COVID patients. After centrifuging freshly collected samples, the hematocrit fraction of each sample was retained and incubated for 30 minutes at room temperature with the two fluorescent markers, CD62P (PE-conjugated) (platelet surface P-selectin) (IM1759U, Beckman Coulter, Brea, CA) and PAC-1 (FITC-conjugated) (340507, BD Biosciences, San Jose, CA). 10 mL of each exposed sample was placed on microscope slide and viewed using a 63x oil objective. Stage 1, with minimally activated platelets, seen as small round platelets with a few pseudopodia, seen as healthy/control platelets that progresses to Stage 4, with egg-shaped platelets, indicative of spreading and the beginning of clumping (with permission from the CC-BY publication 28 ).

There are many reasons why we might anticipate that platelet activation in chronic inflammatory diseases might have many origins, not least the plethora of receptors on platelets that can be activated individually by numerous circulating inflammatory molecules (see Fig. 2 and Table 1 for a basic list of the types of membrane receptors and their functions). Platelets are mostly seen as participating “only” in clot formation during wound healing, but they are actually most important as signaling entities in immuno-thrombosis. Signaling through different receptors can lead to different degrees of platelet activation; however, when a platelet is activated, it cannot return to its original state. Different diseases and even individuals with the same disease have a unique combination and concentration of inflammatory molecules in circulation. It would only need a few of these molecules to bind to any one or more of the various receptors on platelets, to cause platelet hyperactivation. The exact nature of the inflammatory molecule trigger may be different, but the end results will be an “outside-in” signaling activation that, in turn, results in “inside-out” platelet signaling and platelet hyperactivation. This is therefore a case where platelet activation and the concept of “cause and result” should be discussed with care. Nonetheless, microscopy imaging might be particularly useful in studying platelet hyperactivation, as the preparation methods we employ in our research allows for minimal handling of samples and short timeframes between obtaining the sample and doing the analysis. Fig. 3 shows examples of microscopy imaging of platelets from control and long COVID samples.

**Table 1:** Receptor types found on the membranes of platelets and their various functions. For a discussion see 37

Receptor type	Membrane receptor and function	References
Receptors associated with antigen presentation	• CD40 and CD40L (CD154, also known as CD40 ligand) - is a member of the tumor necrosis factor (TNF) family and CD40-mediated platelet activation is well known in the development of thrombosis, inflammation, and atherosclerosis. Human platelets carry preformed CD40L molecules, which rapidly appear on the platelet surface following stimulation by thrombin. CD40L is on the platelet surface for a short time and does participate in an immune reaction, although mainly by being released in soluble form and being a cytokine and not so much a receptor when the strict definition of a receptor is considered. • Major histocompatibility complex (MHC) class I - MHC (class I) is present on platelets, and platelets directly activate naive T cells in a platelet MHC class I-dependent manner. • Toll like receptors (TLRs)—TLR4 has a prominent functional impact on platelet activity, hemostasis, and thrombosis. • Fc receptor for IgG, [FcγRIIa or CD32])—human platelets express FcγRIIa, the low-affinity receptor for the constant fragment (Fc) of immunoglobulin (Ig) G that is also found on neutrophils, monocytes, and macrophages. Engagement by circulating ligands results in immune complexes that further triggers intracellular signaling events that lead to platelet activation and aggregation. An example of such a complex may be FcγRIIa/Integrin αIIbβ3 that might bind immunoglobulin. FcγRIIa may also be associated with GPIb-IX-V. • Complement receptors—platelets contain complement factors and bear complement receptors.	123 124 125 126 127 128 129 130 131 132
Activation and modulating platelet receptors	• CD63—this is a dense granule and lysosome membrane glycoprotein. After platelet activation and granule exocytosis, CD63 translocates to the platelet membrane, where it colocalizes with the αIIbβ3-CD9 complex and is incorporated in the cytoskeleton. This allows platelet interactions with other cells, for example, neutrophils. Both CD63 and CD9 are members of the tetraspanin superfamily of integral membrane proteins that functions as signaling complexes. • Glycoprotein VI[GPVI])—this receptor also signals through a immunoreceptor tyrosine-based activation motif, and is involved in platelet activation. It is a platelet-specific transmembrane type I receptor that non-covalently associates with the immunoreceptor tyrosine-based activation motif (ITAM) containing Fc receptor (FcR) γ-chain in the plasma membrane. It is the main collagen receptor. Activation leads to stable platelet adhesion and degranulation of platelet granules. • C-type-lectin-like receptor (CLEC2)—CLEC-2 is a C-type lectin-like type II transmembrane receptor and is involved in platelet aggregate stabilization. It uses a similar signaling pathway as the GPVI/FcRγ-(chain) complex but it involves tyrosine phosphorylation of only a single cytoplasmic YXXL motif.	133 134 135 136 137 138 139 140 141 142
Adhesion receptors	• GPIb-IX-V (sometimes also written as GPIb-V–IX or GP1b-IX)—This receptor can be classified as both an adhesion and a major signaling receptor, expressed on the surface of circulating platelets. It is composed of four subunits: GPIbα, GPIbβ, GPV, and GPIX. GPIbα and GPIbβ are linked by disulfide bridges, while the GPV and GPIX associate non-covalently with the complex. GPIbα subunit bears the binding site for von Willebrand factor (vWF), leukocyte integrin αMβ2, P-selectin, and the coagulation factors thrombin, and other factors like XI and XII, and Mac-1. GPIb-IX-V may also be associated with FcRγ and with FcγRIIa. • CD147—The extracellular matrix metalloproteinase (CD147) is a member of the immunoglobulin superfamily and is also known as EMMPRIN, localized to the open canalicular system (OCS), and potentially within the α-granules, as its stimulated expression coincided with CD62P release to the platelet surface. Its expression is upregulated in the coronary circulation in patients with stable coronary disease. Its expression is upregulated in response to platelet agonists, including ADP, collagen and thrombin. • P-selectin—It is an important adhesion receptor, and is an adhesion molecule (CD62P) component of the platelet membrane. After platelet activation, P-selectin is translocated from intracellular α granules to the external membrane, therefore α-degranulation results in increased surface expression of P-selectin. Shedding of P-selectin from the platelet membrane can also happen as released soluble ectodomain fragments that are also detectable in plasma. Plasma levels of soluble P-selectin (sP-selectin) are often used to demonstrate platelet activation. P-selectin mediates rolling of platelets and leukocytes on activated endothelial cells. Thrombin may also cause exposure of activated P-selectin.	131 142 143 144 145 146 147 148 149 150 151 152 153 154 155
Intergrins	• Integrin αVβ3—this is the vitronectin receptor. Vitronectin is a glycoprotein of the hemopexin family. It is also known to assemble fibronectin fibrils on platelets and mediates cell adhesion to the extracellular matrix. • Integrin αIIbβ3—this is a receptor for fibrinogen and von Willebrand Factor (VWF), and activation of this receptor induces platelet aggregation and may play an important role in platelet spreading. Upon platelet activation, inside-out signaling pathways increase the affinity of αIIbβ3 for fibrinogen and other ligands. *Inside-out signaling* therefore facilitates and initiates the conformational changes responsible for ligand binding. Ligand binding and integrin clustering follows, resulting *outside-in signaling* , which initiates and amplifies cellular events driving essential platelet processes such as spreading, thrombus consolidation, and clot retraction. • Integrin α2β1—this is a collagen receptor and collagen binds directly or indirectly to both α2β1 (and αIIbβ3, via VWF). • One of the main platelet activators is also thrombin that causes exposure of activated αIIbβ3, αvβ3, α2β1 (also P-selectin as mentioned in the previous section).	137 155 156 157 158 159 160 161
G-coupled receptors	• Protease-activated receptors (PAR1 and PAR4)—platelets contain both these receptors and PAR1 and PAR4 acts as a dual receptor system for responding to thrombin as ligand. Both PAR1 and PAR4 also signal via ADP. • ADP receptors, P2Y1 and P2Y12—they are involved in platelet activation and aggregation. • The thromboxane A2 (TXA2) receptor—it exists as two isoforms, TPα and TPβ, differing only in their C-terminal region.	162 163 164 165 166 167 168 169 170 171

## Ten Things We know about the Fibrinaloid Microclots That Can Be Observed in Chronic, Inflammatory Diseases

**Fig. 4:**
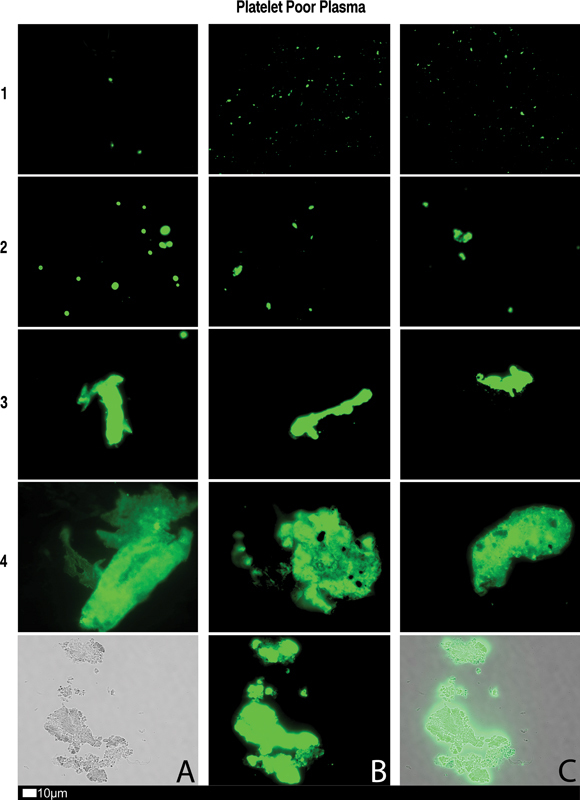
Fluorescence microscopy showing microclots in platelet poor plasma (PPP) with representative examples of the different stages of different stages of microclot formation. Stored or freshly prepared platelet poor plasma samples were exposed to Thioflavin T (ThT), a fluorogenic dye that binds to amyloid protein. 15 A final concentration of 0.005 mM was used (Sigma-Aldrich, St. Louis, MO). Plasma was exposed for 30 minutes (protected from light) at room temperature, whereafter 3 µL stained PPP was placed on a glass slide and covered with a coverslip. Stage 1 shows minimal microclot formation in healthy/control PPP which progresses to the presence of the severe microclotting Stage 4. Bottom row represents examples of stage 4 microclots using ( **A** ) bright-field microscopy, ( **B** ) fluorescence microscopy, and ( **C** ) an overlay of fluorescence and bright-field microscopy (with permission from the CC-BY publication 28 ).

**Fig. 5:**
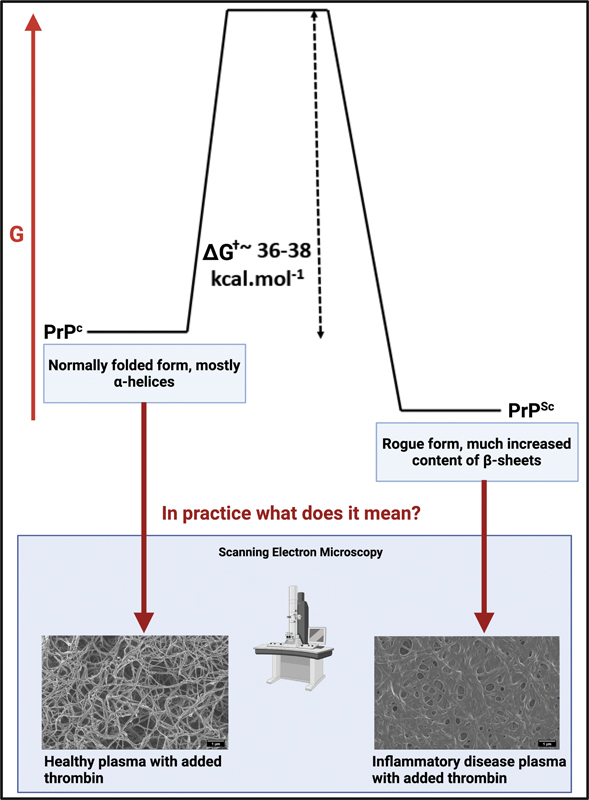
Protein–protein interactions may result in protein misfolding and have been shown to cause amyloidogenic changes to all kinds of proteins. This is illustrated in the upper part for the Prion protein PrP. The lower part shows electron micrographs of fibrin clots. Adapted in part from the CC-BY publications. 15 17 64 We also note that the spike protein is itself amyloidogenic. 172 Figure created by authors using Biorender.com.

We think it will be helpful to rehearse some of the known facts about fibrinaloid microclot formation in the form of a “ten things” 63 style ( Table 2 ); also please see Fig. 4 for examples of fluorescence microscopy imaging of microclots. Many proteins can adopt a more thermodynamically stable microstate with no change in the primary structure (sequence), in which the more stable contains an ordered β-sheet “amyloid” structure 15 (see Fig. 5 ). Normally, however, it is present in a less stable state that is kinetically more accessible during and following its synthesis. The more stable (labeled PrPSc) is separated from the initial state (PrPC) via a large energy barrier. This is true for amyloid proteins generally and is illustrated here for classical prion proteins. 17 64

**Table 2:** The “big picture” of microclots and ideas for future research related to microclots

Fibrinaloid microclots are widely present in all chronic, inflammatory diseases studied to date, and to a much greater extent than in ‘healthy’ controls.
These diseases include Alzheimer's, Parkinson's, rheumatoid arthritis, and infection with SARS-CoV-2 leading to acute or long COVID-19.
They can be stained with fluorogenic dyes such as thioflavin T or the Amytracker dyes.
They are commonly in the size range 2–200 μm.
They are comparatively resistant to the normal processes of fibrinolysis; some are even resistant to trypsin.
Their extent is often related to the severity of various diseases.
They can be induced in vitro (in both whole blood and platelet poor plasma) with a variety of substances, including bacterial lipopolysaccharide, lipoteichoic acid, 17-b-oestradiol, and SARS-CoV-2 S1 spike protein.
In some cases (especially SARS-Cov-2 infection) they are prevalent even without the addition of thrombin.
The diameter of the fibers can vary fairly considerably.
They can exhibit considerable structural (and even spectral) heterogeneity, reflecting the molecules that were bound to the fibrinogen before polymerization.

Abbreviations: COVID-19, coronavirus disease 2019; SARS-Cov-2, severe acute respiratory syndrome coronavirus 2.

**Fig. 6:**
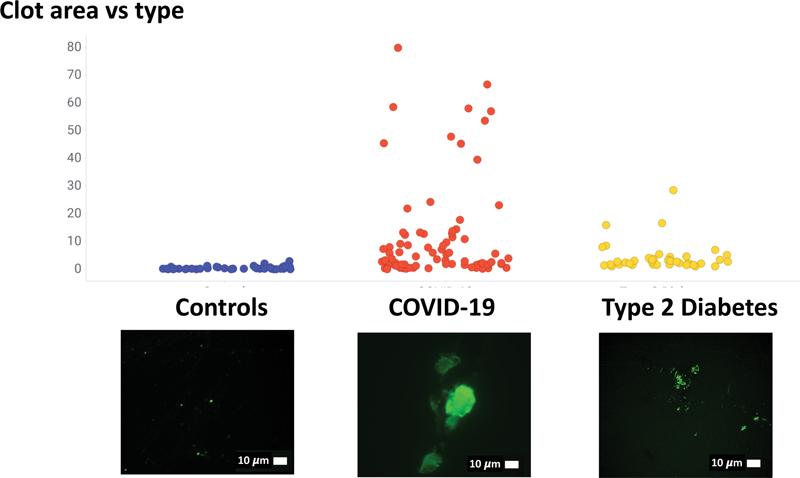
Comparison of microclot area in healthy participants, and participants with Type 2 diabetes and acute COVID-19. (Raw data reanalyzed, and available in 26 ).

**Fig. 7:**
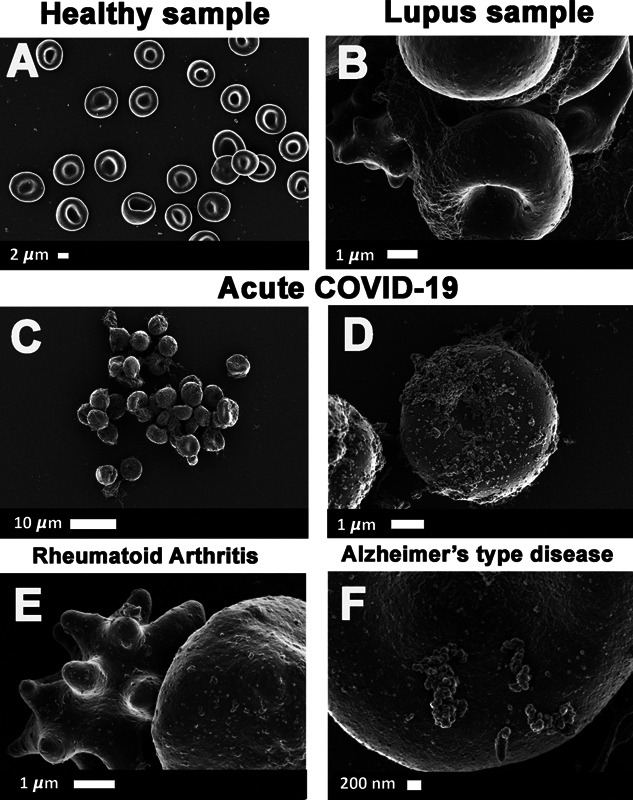
( **A to F** ) Whole blood scanning electron microscopy of micrograph data previously collected in published studies. ( **A** ) A few plasma deposits are discernible within the blood sample of a healthy participant 173 ; ( **B** ) systemic lupus erythematosus 174 ; ( **C and D** ) acute COVID-19 during the initial wave in 2020 30 ; ( **E** ) rheumatoid Arthritis 175 ; ( **F** ) Alzheimer's-type disease. 176

Fig. 6 offers a comparative examination of microclot areas reanalyzing fluorescence microscopy data, previously published, 26 extending our prior analysis to include healthy participants, individuals with T2DM, and those currently experiencing acute COVID-19. Reexamining some of our previously collected micrograph data from previously published papers, we also provide evidence that microclots are visible in scanning electron microscopy (SEM) of whole blood samples. Fig. 7 showcases a selection of SEM micrographs of healthy whole blood and conditions where clotting pathologies are well-known. In Fig. 7A , a few plasma deposits (microclots) are discernible among or on the erythrocytes, while Fig. 7B to F show whole blood SEM micrographs of samples from an individual diagnosed with systemic lupus erythematosus ( Fig. 7B ), acute COVID-19 during the initial wave in 2020 ( Figs. 7C and D ), rheumatoid arthritis ( Fig. 7E ), and a sample from a patient diagnosed with Alzheimer's-type dementia ( Fig. 7F ). We only realized that these deposits were of significant importance during the last few years.

## Variation in Microclot Properties

We remain relatively ignorant of the details of precisely what governs fibrin self-assembly following fibrinopeptide release (e.g., why it stops at a certain and nonconstant fiber diameter, even in health, 65 and why it can vary with disease 66 ). One thing we do know, however, is that microclots covary with (and in our view are thus largely responsible for) the severity of disease in both acute and long COVID 67 and in fact contribute to widespread thrombotic endothelialitis in long COVID. 62

## What Kind of Molecules can Promote Fibrinaloid Formation?

Since the early discoveries that molecules such as oestrogens 68 69 could induce the formation during clotting of “dense matted deposits” (that we now refer to as fibrinaloid microclots, because of their amyloid character), we have also found that low concentrations of molecules such as bacterial lipopolysaccharide, 14 lipoteichoic acid, 21 serum amyloid A, 70 and SARS-CoV-2 S1 spike protein 34 can also do so. These dense matted deposits could be induced using both whole blood and platelet-poor plasma. It is moderately to highly unlikely that they are binding in the same locations as each other (the binding site of spike is known 71 ), and thus, the morphology of the clots they induce will be the same. In a similar vein, nonamyloid substances can also bind to and induce the aggregation of established amyloid proteins such as bacterial DNA promoting tau 72 or β-amyloid 73 aggregation.

## Consequences of Microclot Formation

The fibrinaloid microclots that we discovered and that can be induced my miniscule amounts of molecules such as bacterial LPS or the SARS-CoV-2 spike protein are resistant to normal fibrinolysis for a least two reasons: (1) amyloid-type proteins are inherently more resistant to proteolysis 35 74 because of their crossed-β structure, 15 75 76 and (2) because they also entrap inhibitors of normal proteolysis such as α2-antiplasmin and plasminogen activator inhibitor 1 (PAI-1). 74 Consequently, they are able to block microcapillaries, leading to tissue ischemia and hypoxia, from which the great majority of symptoms can be seen to follow. 17 77 Importantly, our analysis provides a mechanistic link between external trigger events and the pathologies of present interest, as well as a candidate set of targets for treatment.

## Anticipating Disease Diagnosis and Prognosis from Microclot Measurements, as Part of Disentangling Their Differences

To date, our microscopy imaging and analyses have mostly been semiquantitative, showing the existence of different microclots in various diseases. As with much of modern, postgenomic biology, 78 especially that influenced by “deep learning” 79 , it is time to move from a “discriminative” strategy. We suggest a “generative” strategy, in which we seek to solve the “inverse problem” (as in 80 ) by using the observables (microclot properties) to infer their “cause” (i.e., the diseases with which they are associated). The observables include the distribution of clot sizes and morphologies, 26 81 the diameter of individual fibers, 82 83 their ability to cross-seed other amyloidogenic proteins, 84 85 86 87 88 89 the spectral properties of the different stains when attached to fibers, 90 91 92 93 94 95 96 the susceptibility of the clots to proteolysis by different proteases, 35 97 and the extent to which they are naturally present in platelet-poor plasma versus being induced by the in vitro addition of thrombin. 17 34 98

## The Power of Multivariate Data, and How the Manner of Fibrinogen Clotting Effects Dimensionality Reduction of the Various Plasma 'omes

Each property of an individual example in a system of interest can be seen as an element of a vector describing that system or as a dimension in multidimensional space. 99 100 101 When we have a series of properties of both a sample “as a whole” and indeed of individual objects therein, it becomes increasingly easy to discriminate them. 100 Care is needed, however, 102 since even comparatively small random variations in normal distributions can appear significant in individual dimensions when there are many to choose from, even in “unsupervised” methods (in which class membership, such as a particular disease, is not known 103 ). In favorable cases (e.g., 104 105 ), though, these are entirely sufficient. Supervised methods, in which a model is “trained” to predict an output set of properties (such as a particular disease) from “input” variables such as omics data, are even more prone to overtraining and other kinds of bias. 102 The solution here (e.g., 106 ) is to assess any predictions taken from such a mathematical model using separate “validation” examples whose class membership or other output properties are known but which are not used in the construction of the model.

Another modern trend is to recognize that the amount of unlabeled data available normally vastly exceeds that of labeled data and that such data can be used in the training of a supervised model; these methods are known as “semisupervised.” They have become preeminent in deep learning models 79 based on variational autoencoders 107 108 109 and transformers, 110 111 especially in natural language 112 113 and image processing. 114 115 116

In variational autoencoders, one essentially clusters input examples into a (much) lower dimensional space. This still allows considerable discrimination; however, even a normalized vector of just 20 in which an individual may be in the upper or lower half admits 2 ^20^ (approximately 1 million) possibilities. 109 117 In the case of microclots, we consider that (distributions in) the many 1,000s of individual metabolites 118 119 120 and proteins 121 in serum or plasma can potentially each affect the size and shape of the microclots. This “harvesting” of all the molecules to which the fibrinogen is exposed, and which then determines how it polymerizes, effectively concentrates the vast numbers of metabolites, proteins, and even transcripts into a smaller number of dimensions; the microclots essentially act as a surrogate for the metabolome, proteome, and transcriptome present in the plasma at the time of clotting. Consequently, we are optimistic that an analysis of the detailed morphological and spectral properties of microclots will indeed serve to discriminate, possibly quite finely, individuals with different diseases.

## Conclusion

In our previous work, we have focused more or less qualitatively on the presence of fibrinaloid microclots that we discovered using microscopy imaging while recognizing that some of their measurable properties differ in various conditions or diseases. Within the terminology of the deep learning agenda, 79 this is to be seen as a “discriminative” approach. The opposite strategy, amounting to the solution of the inverse problem, is referred to in general as a “generative” strategy. Here the aim, now clearly worthwhile, is to develop and exploit a more quantitative analysis in the prediction of diseased states, and the success of any treatment both in modifying the microclot properties and in curing or ameliorating the diseases. Various laboratories in the United States, United Kingdom, and Germany have successfully implemented microclot imaging and will also be publishing their results shortly. We have been using and described here, fluorescence microscopy imaging (and also briefly SEM methods) to visualize microclot presence and platelet hyperactivation. In addition, we are also developing imaging flow cytometry methods that may, in future, direct a meaningful translational outcome that could guide treatment options. We also suggest that automation of our microscopy imaging using software modalities like the MetaSystems platform ( https://metasystems-international.com/ ), might be particularly useful for unbiased quantitative analysis of microscopy imaging of both platelets and microclots. Other research teams are developing innovative strategies that integrate real-time one-dimensional-imaging fluorescence with deformability cytometry within a single instrument, denoted as RT-FDC. 122 RT-DC techniques are also currently being adapted to study and characterize the mechanical classification of microclots in whole blood, at rates of several hundred cells per second. We believe that the research agenda, using various novel methods, as set out here, will make the enterprise of unbiased quantitative analysis of microclots, worthwhile. We suggest that researchers direct increased attention toward a previously disregarded fraction of the blood sample. Microclots found in platelet-poor plasma could hold a significant diagnostic value for clotting-related issues in inflammatory diseases and other similar conditions, including postviral syndromes.
